# A modified tetra-bite-on-bite biopsy technique under cholangioscopic guidance for biliary disease

**DOI:** 10.1055/a-2723-1724

**Published:** 2025-11-04

**Authors:** Takeshi Ogura, Junichi Nakamura, Takafumi Kanadani, Kimi Bessho, Hiroki Nishikawa

**Affiliations:** 1 Pancreatobiliary Advanced Medical Center, Osaka Medical and Pharmaceutical University Hospital, Osaka, Japan; 2 13010 Endoscopy Center, Osaka Medical and Pharmaceutical University, Osaka, Japan; 3 2nd Department of Internal Medicine, Osaka Medical and Pharmaceutical University, Osaka, Japan

Bile duct strictures can occur due to various diseases, such as cholangiocarcinoma or primary sclerosing cholangitis. To make a differential diagnosis, obtaining tissue from the target lesion is clinically important. Cholangioscopic-guided biopsy may be useful, because biopsy of the target lesion can be performed under direct visualization 1 2 . However, the amount of obtained tissue may be small. Therefore, efforts to obtain larger tissue samples are needed. The bite-on-bite biopsy technique can obtain tissue from the submucosal layer, and this technique has already been reported for diagnosing submucosal tumors. Recently, the bite-on-bite biopsy technique was compared to the standard biopsy technique for indeterminate biliary strictures, but this technique was not significantly superior to the standard technique 3 . However, during the bite-on-bite biopsy technique, a small amount of tissue is obtained because each tissue sample may be separately evaluated, although tissue can be obtained from deeper sites. To overcome this, we developed a modified tetra-bite-on-bite (mBBB) technique. Technical tips for the mBBB technique are presented.

Biliary cannulation was attempted, and contrast medium was injected. On cholangiography, hepatic hilar stenosis was observed ( Fig. 1 ). Then, the cholangioscope was inserted into the biliary system. A papillary lesion was observed at the hepatic hilar region ( Fig. 2 ). To obtain sufficient tissue, mBBB was attempted. First, a forceps biopsy device (SPY-Bite MAX, Boston Scientific) was advanced to the tumor lesion through the cholangioscope channel. Subsequently, the first biopsy bite was performed ( Fig. 3 ). Without removing the forceps biopsy device from the cholangioscope, a second biopsy was taken at the same site ( Fig. 4 ). A third biopsy was then performed in the same manner. By performing consecutive biopsies at the same location without removing the forceps, en bloc tissue collection was successfully achieved without any adverse events ( Fig. 5 ; Video 1 ). Histological examination showed adenocarcinoma.

**Fig. 1:**
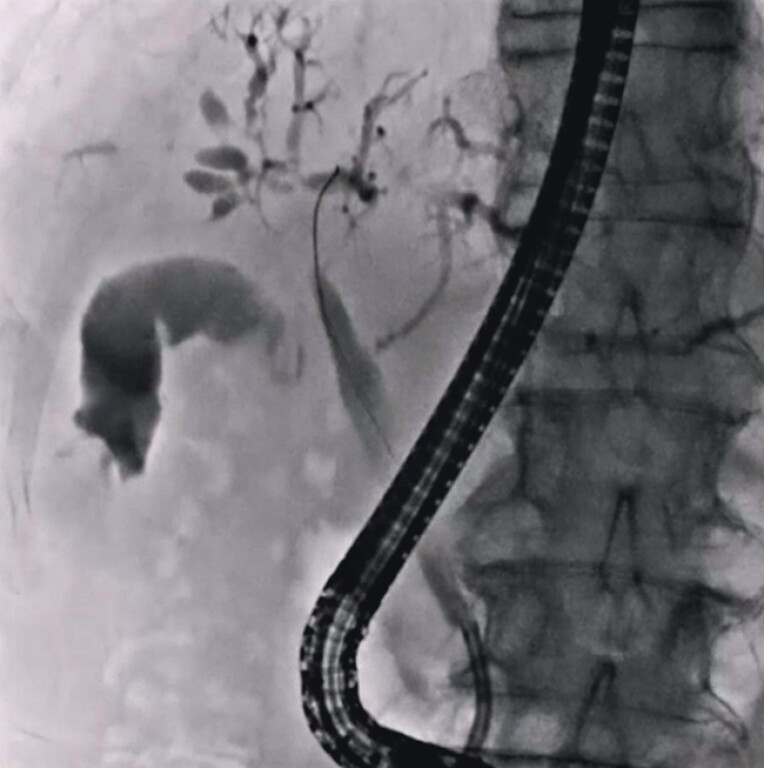
On cholangiography, hepatic hilar stenosis is observed.

**Fig. 2:**
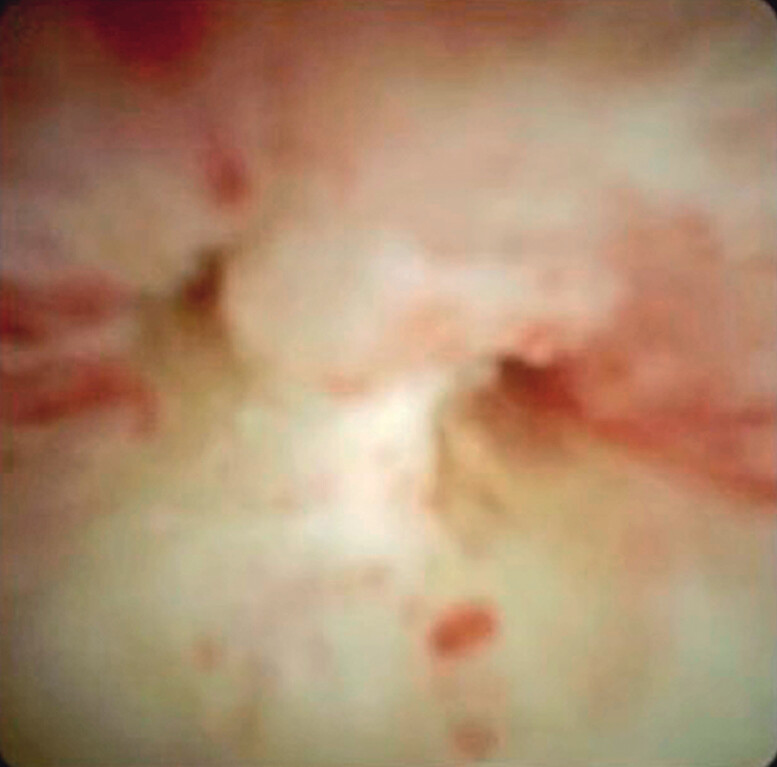
A papillary lesion is observed in the hepatic hilar region.

**Fig. 3:**
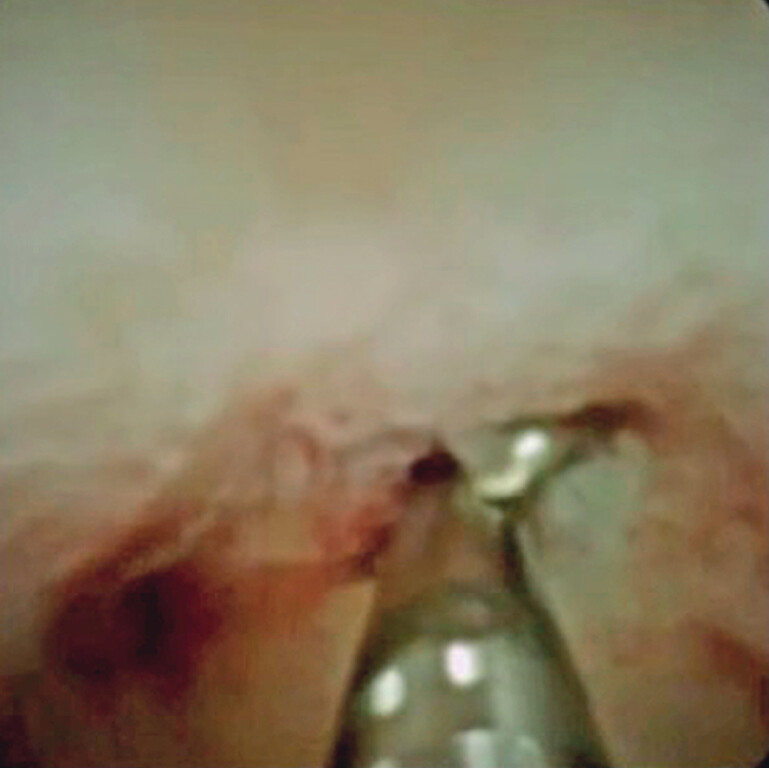
The first biopsy bite was performed.

**Fig. 4:**
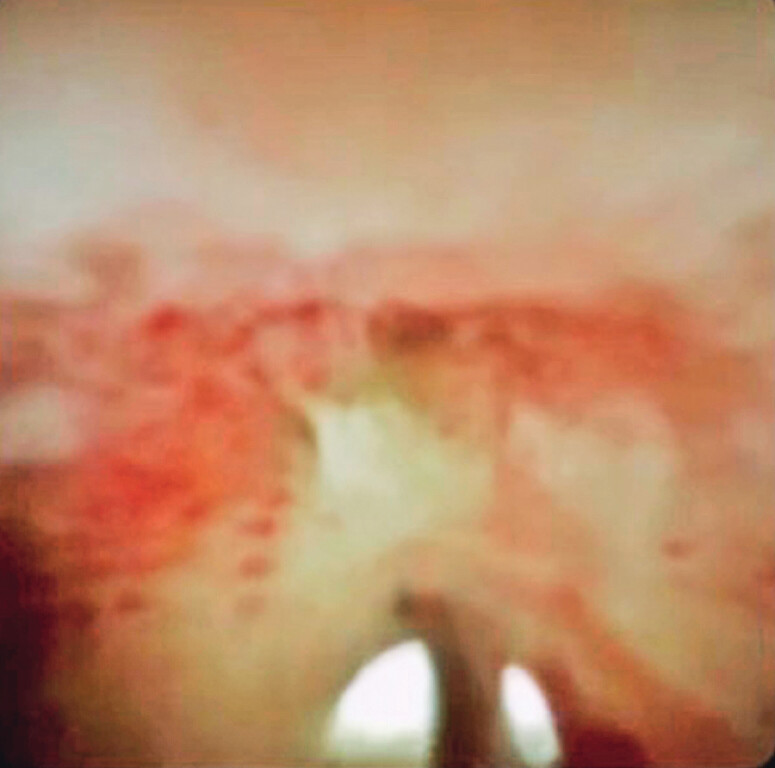
Without removing the forceps biopsy device from the cholangioscope, a second biopsy is taken at the same site.

**Fig. 5:**
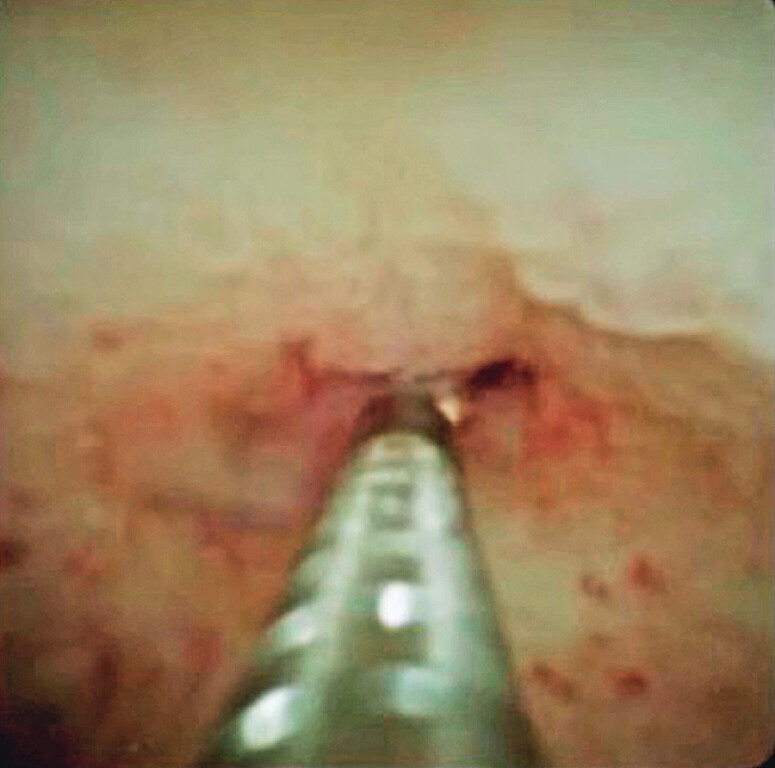
A third biopsy is then performed in the same manner.

A modified tetra-bite-on-bite technique.Video 1

In conclusion, mBBB might be useful to obtain larger tissue samples, although further evaluation is needed.

Endoscopy_UCTN_Code_TTT_1AR_2AD

## Notes

Endoscopy E-Videos https://eref.thieme.de/e-videos
*E-Videos* is an open access online section of the journal *Endoscopy* , reporting on interesting cases and new techniques in gastroenterological endoscopy. All papers include a high-quality video and are published with a Creative Commons CC-BY license. Endoscopy E-Videos qualify for HINARI discounts and waivers and eligibility is automatically checked during the submission process. We grant 100% waivers to articles whose corresponding authors are based in Group A countries and 50% waivers to those who are based in Group B countries as classified by Research4Life (see: https://www.research4life.org/access/eligibility/ ). This section has its own submission website at https://mc.manuscriptcentral.com/e-videos .
